# Effect of coupled reduced irrigation and nitrogen fertilizer on soil mite community composition in a wheat field

**DOI:** 10.1002/ece3.5638

**Published:** 2019-09-18

**Authors:** Chunyan Zheng, Fang Ouyang, Xianghui Liu, Junhua Ma, Fenghua Zhao, Zhu Ouyang, Feng Ge

**Affiliations:** ^1^ State Key Laboratory of Integrated Management of Pest and Rodents Institute of Zoology Chinese Academy of Sciences Beijing China; ^2^ Yucheng Station Key Lab of Ecosystem Network Observation and Modeling Institute of Geographic Sciences and Natural Resources Research Chinese Academy of Sciences Beijing China

**Keywords:** community composition, functional guild, indirect effect, mite, reduced addition

## Abstract

Groundwater and nitrogen fertilizer overuse severely threatens crop productions; thus, current ecological agriculture requires low irrigation and nitrogen fertilizer inputs. The effects of combined reduced irrigation and nitrogen fertilizer addition on soil organism (e.g., mite) community and biodiversity remain poorly understood. We analyzed soil mite community composition, wheat grain yield, and soil characteristics in a 10‐year manipulation experiment with two levels of irrigation (reduced and conventional irrigation) and five nitrogen fertilizer levels (0, 70, 140, 210, and 280 kg N/ha). Reduced irrigation (20% reduction, from 280 to 220 mm) and nitrogen fertilizer (25% reduction, from 280 to 210 kg N/ha) addition did not significantly influence soil mite community and wheat yield. The relative abundances of fungivores and predators showed negative quadratic relationships with wheat yield, while that of plant parasites showed a positive relationship. The relationships between soil mite trophic groups and wheat yield revealed that we can evaluate the impacts of reduced irrigation and nitrogen fertilizer addition from the perspective of soil fauna. Soil mite community composition was altered by soil abiotic factors prior to reduced irrigation and nitrogen fertilizer addition. Overall, moderate reductions of irrigation and nitrogen fertilizer may not threaten to soil mite community and diversity or decrease crop production; in contrast, such reductions will benefit mite community development and the sustainable agriculture.

## Graphical abstract

Moderate reductions of IR and N fertilizer may not decrease crop production or threaten to soil organism community and diversity. On the contrary, it will benefit to decrease agricultural inputs and maintain the sustainable development of agriculture.

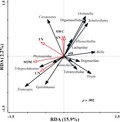

## 1 INTRODUCTION

Soil mites, the most abundant microarthropods in soils, occupy multiple trophic levels in the soil food web and play critical roles in agroecosystems (Coleman, 2013). These mites are classified into fungivores, plant parasites, omnivores, and predators according to their feed habits (Arjen, Gerard, Wim, Xavier, & Jack, 2016; Coleman & Whitman, 2005), and different trophic groups contribute directly or indirectly to plant growth by taking roles in the processes of soil nutrients cycling, soil formation, and pest control (Eisenhauer, 2010; Fathipour & Maleknia, 2016; Wardle et al., 2004). Fungivores and plant parasites contribute to soil nutrient cycling by regulating microbial activity and digesting cell wall material to influence nutrients release and activation (Kaneko, McLean, & Parkinson, 1998; Schneider & Maraun, 2005; Siepel & Maaskamp, 1994). Some mite groups (e.g., fungivores and omnivores) contribute to soil formation and aggregate stability through burrowing, through fragmenting organic materials, and through producing feces and feeding activity (Duchicela, Sullivan, Bontti, Bever, & Wan, 2013; Maaß, Caruso, & Rillig, 2015; Siepel & Maaskamp, 1994; Soong & Nielsen, 2016). In addition, many predators have been studied because of their potential biological control value in agroecosystem achieved by reducing the usage of pesticides (Carrillo, Moraes, & Peña, 2015; Fathipour & Maleknia, 2016). Based on these contributions, changes in the community diversity and composition of mite trophic groups can be expected to impact crop yields and ecosystem productivity. Furthermore, the composition of the mite community can be affected by soil environmental characteristics (e.g., soil organic matter, pH, and available phosphorus) via changes in exogenous inputs (Cao et al., 2011; Melguizo‐Ruiz et al., 2017; Wang, Tan, Fan, Ruan, & Zheng, 2015). Therefore, monitoring soil mites responses may help us better understand the effects of different artificial disturbance on agroecosystems.

The North China Plain (NCP) is one of the major agricultural regions in China and provides approximately 80% of the nation's wheat (NBS, 2018). From 1978 to 2018, as a result of intensive agricultural management (e.g., mechanization, irrigation, and mineral fertilization), the annual wheat yield increased by 148% (from 54 to 134 million tons) and the average grain yield per unit area increased by 198% (from 1.85 to 5.48 t/ha) in China (NBS, 2018). However, side effects following increased crop yields include groundwater overuse and excessive nitrogen application. For example, in the NCP, annual precipitation is uneven and insufficient during the wheat grown season (Meng et al., 2012). To achieve high grain yields, additional water has been applied, which has resulted in a rapid decline in the groundwater table at an average rate of approximately 1 m per year in recent years (IGSNRR, 2016) and restricted sustainable agricultural development (Zhang et al., 2004). In addition to water shortages, excessive N application has also caused a series of environmental problems, including increased greenhouse gas emission, soil acidification, and biodiversity loss (Bardgett & Wardle, 2010; Guo et al., 2010; Tan et al., 2017). The amount of fertilizer applied is far greater than the wheat growth demand (325 kg N/ha; Ju et al., 2009), while the recommended nitrogen rate for wheat in the winter wheat–summer maize rotation systems in the NCP is 203 to 230 kg/ha (Liu et al., 2016). Therefore, there is an urgent need to identify adequate combinations of reduced irrigation and nitrogen application to ensure sufficient yield with relatively lower environmental costs for the government and researchers (Chen et al., 2014; Ju et al., 2009).

Water and nitrogen are two of limiting factors of crop growth, and changes in the levels of water and nitrogen applied to soil could influence ecosystem function and organisms community composition in agroecosystems (Liang et al., 2009; Paungfoo‐Lonhienne et al., 2015; Zhou, Wang, Zheng, Jiang, & Luo, 2017). Soil organisms (including microorganisms, nematodes, collembolas, and mites) play crucial roles in nutrients cycling, crop growth, and ecosystem processes. Therefore, it is important to understand the different response of soil organisms to water and nitrogen management in order to predict their sensitivity to agricultural production measures. Changes in water and nitrogen fertilizer addition may affect soil organism communities through different mechanisms. Although many studies had been conducted to evaluate the effects of water and nitrogen fertilizer addition on soil organism community, many variations and inconsistencies can be found in the literature (Melguizo‐Ruiz et al., 2017; Sun et al., 2017). For example, elevated water addition increased soil fungi and decreased soil bacteria, while increased nitrogen fertilizer addition had the opposite effects because water addition led to significant changes in soil characteristics (Ma et al., 2016). Moreover, the effects of water and nitrogen fertilizer addition on the soil nematode community were complex and lack consistency. For instance, water addition enhanced the abundance and species richness of soil nematodes, but nitrogen fertilizer addition reduced their generic richness (Song et al., 2016), and the effect of different levels of nitrogen fertilizer addition on soil nematode also fluctuated (Chen et al., 2019; Song et al., 2015). In addition, although several studies have evaluated the combined effects of water and nitrogen fertilizer addition on soil organisms (Li et al., 2016; Zhang et al., 2018), few studies have researched the response of soil mites to changes in water and nitrogen fertilizer application, with the exception of two studies in forest ecosystem (Lindberg & Persson, 2004; Melguizo‐Ruiz et al., 2017).

To investigate the hypothesis that potential changes in soil physicochemical properties caused by reduced irrigation and nitrogen fertilizer addition affect mite community composition and then the wheat yield, a long‐term field manipulative experiment with reduced irrigation and nitrogen fertilizer addition treatments was conducted in a wheat farmland ecosystem. Monitoring soil mites responses to reduced irrigation and nitrogen fertilizer addition may help us better understand the effects of different artificial disturbance on agroecosystems. The objectives of the present study were (a) to evaluate the changes in wheat yield and mite community composition under reduced irrigation and nitrogen fertilizer addition treatments, (b) to identify the main drivers of the soil mite community (e.g., reduced irrigation addition, reduced nitrogen fertilizer addition, or soil physicochemical properties), and (c) to examine how potential changes in mite community composition affect wheat yield.

## 2 MATERIALS AND METHODS

### 2.1 Experimental setup

A field experiment was conducted at the Yucheng Comprehensive Experimental Station in southwestern Shandong Province, China (36°57′N, 116°36′E, 20 m elevation). The experimental site is located in a typical temperate semiarid zone with a mean annual temperature of 13.3°C and an annual rainfall of 559.8 mm according to long‐term observations (1998–2015; Zhao, Li, Ai, Li, & Gu, 2017). The soil is classified as a Calcaric Fluvisols according to the FAO‐Uneson system and contains 12.9% sand, 65.1% silt, and 22.0% clay. At the beginning of the experiment, the surface soil (0–20 cm) had a pH of 8.3 (H_2_O 1:2.5) as well as 12.2 g/kg soil organic matter (SOM) and 0.5 g/kg total nitrogen (TN).

The experiment followed a split‐plot design with irrigation and nitrogen fertilizer addition in a wheat and maize rotation system established in 2005. There were ten treatments with the combinations of two levels of irrigation addition and five levels of nitrogen fertilizer, with three replicates for each treatment. The plots were hydrologically isolated with partition walls filled with cement to prevent unexpected species moving between the individual plots. The main plots received the conventional irrigation (CIR) and reduced irrigation (RIR) treatments (80% of CIR), and subplots were randomly assigned to five levels of nitrogen fertilizer addition (0, 70, 140, 210, and 280 kg N/ha in the grown season, denoted N0, N1, N2, N3, and N4, respectively). The CIR and N4 treatments were set according to the typical applications used by local farmers. Irrigation was applied four times over the wheat growing season, and nitrogen fertilizer was added in the form of urea, which was applied prior to wheat sowing (50% of the total dose) and at the jointing stage (50% of the total dose). For all treatments, a total of 120 kg/ha P_2_O_5_ (as Ca(H_2_PO_4_)_2_) and 80 kg/ha K_2_O (as K_2_SO_4_) were applied in each plot before sowing.

### 2.2 Sampling and measurement of grain yields and environmental parameters

Soil samples were collected from each plot before wheat harvest in 2014 and 2015. In each subplot, nine soil cores (2.5 cm diameter) were collected from a depth of 0–20 cm and combined. The soil samples were divided into two subsamples: one was used to extract soil mites, and the other was sieved through a 2‐mm sieve and air‐dried for soil environmental parameters analysis. Wheat yield was evaluated on the day of harvest. Wheat grains were harvested from three areas measuring 1 m^2^ (1 m × 1 m) in each plot and oven‐dried at 60°C, after which the dry weight was determined. Soil environmental parameters were measured and have been described elsewhere (unpublished). Briefly, the soil organic matter (SOM) content was not significantly influenced by reduced irrigation, nitrogen fertilizer addition, or their interaction (generalized linear mixed models [GLMMs], *p* > .05). In contrast, the available nitrogen content (AN) and carbon to N ratio (CN ratio) presented an initial increase followed by a decrease in response to nitrogen fertilizer addition under CIR addition, and the same trend was observed for total nitrogen (TN) content under RIR addition. Furthermore, the soil water content (SWC) decreased from 16.1 ± 0.7% to 10.3 ± 0.9% in the reduced irrigation and nitrogen fertilizer addition treatments. Soil pH decreased significantly in response to the interaction effect of elevated irrigation and nitrogen fertilizer addition, but the decrease was small.

### 2.3 Soil mite extraction and identification

Soil mites were extracted from 500 cm^3^ of fresh soil by modified Tullgren funnels (Wallwork, 1976; Zhu & Zhu, 2015), placed under a 40 W bulb for 48 hr, and preserved in 75% ethanol. The soil mites were mounted on permanent slides using Hoyer's medium and were identified under a binocular dissecting microscope (Olympus CX21FS1). The adult mites were assigned to four trophic groups (fungivore, plant parasite, omnivore, and predator) according to their feeding habits (Badejo, Tian, & Brussaard, 1995; Carrillo et al., 2015; Fathipour & Maleknia, 2016; Maraun, Augustin, Müller, Bässler, & Scheu, 2014; Masan & Halliday, 2014; Moore, Walter, & Hunt, 1988; Palacios‐Vargas, Castaño‐Meneses, & Estrada, 2011; Siepel & Emde, 1993), and nymphs were pooled to one group. The mite population was expressed as individuals per square meter of soil, and the relative abundance of each trophic group was determined.

### 2.4 Statistical analysis

Data analyses were conducted using R software (2018, version 3.5.1, R Foundation for Statistical Computing, https://www.r-project.org/). The data were log‐transformed prior to analyses to improve normality. The effects of reduced irrigation and nitrogen fertilizer addition on response variables (wheat yield, soil mite abundance, and soil mite community composition) were examined using split‐plot ANOVA for each sampling year. Reduced irrigation and nitrogen fertilizer addition, as well as their interactions, were assigned as fixed factors, and irrigation was nested within block as an error term. Additional one‐way ANOVAs with the least significant difference were performed to compare the means among nitrogen fertilizer levels for each irrigation treatment. Regression models were used to determine the relationships between wheat yields and the relative abundances of the four soil mite trophic groups. Mantel tests and partial Mantel tests were used to examine the effects of sampling year, reduced irrigation and nitrogen fertilizer addition, and soil environmental parameters on mite community composition.

Nonmetric multidimensional scaling (NMDS) plots were used to visualize the soil mite community composition variations among samples based on the Bray–Curtis dissimilarity index matrices. A heatmap was created on the basis of each mite taxon abundance using the *pheatmap* package. Redundancy analysis (RDA) was employed to visualize the relationships among soil environmental parameters and soil mite taxa. Stepwise regression analysis was used to identify the factors that could effectively explain the changes in mite community composition in response to soil environmental parameters. The analyses of Mantel test, partial Mantel test, NMDS, and RDA were performed using the *vegan* package. All other figures were generated by SigmaPlot 10.0 (Systat Software Inc.).

## 3 RESULTS

### 3.1 Correlations between reduced irrigation addition, reduced nitrogen fertilizer addition or mites and wheat yields

**Figure 1:**
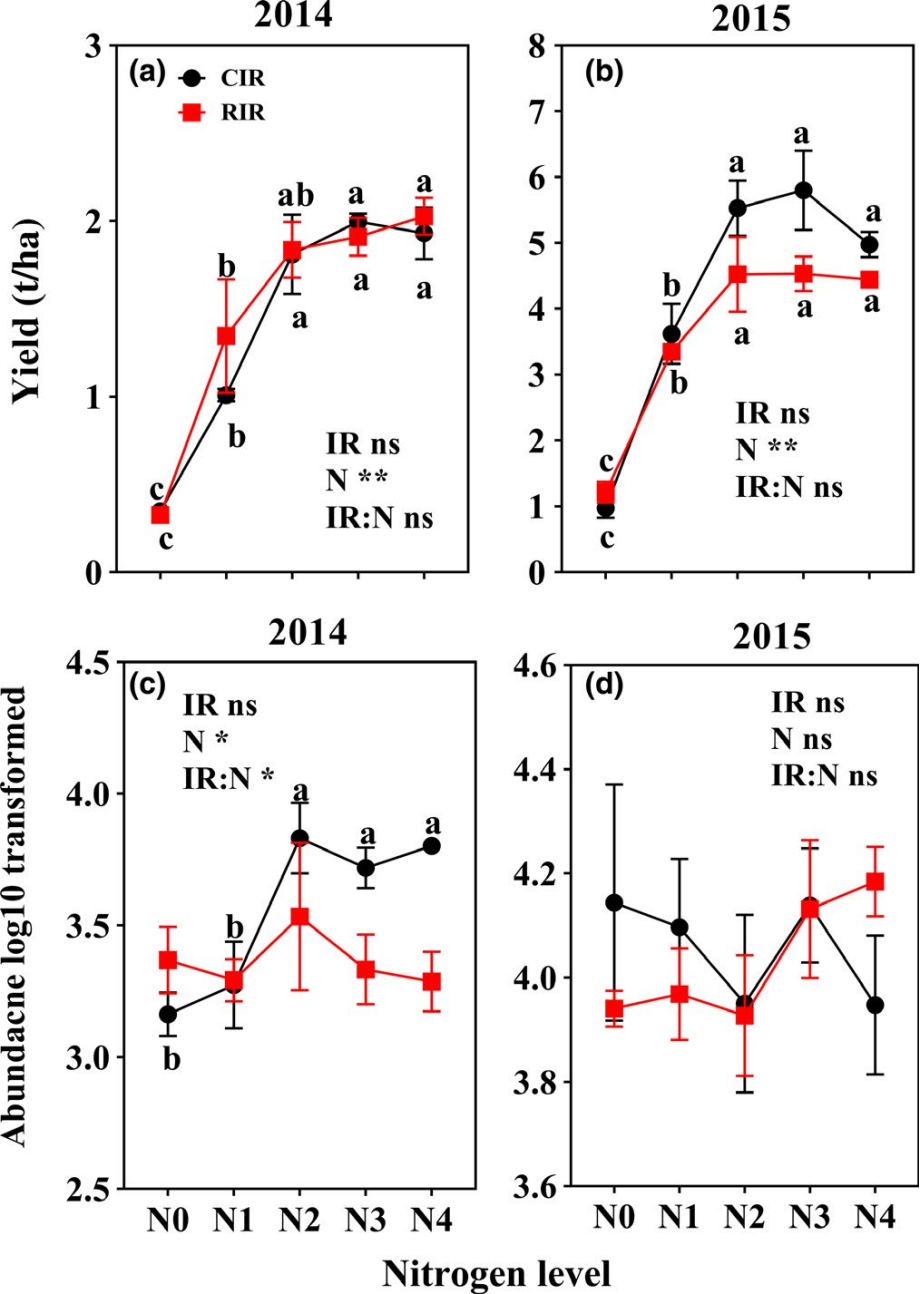
Changes in wheat yields (a and b) and total soil mite population (c and d) under reduced irrigation and nitrogen fertilizer addition treatments (CIR, conventional irrigation; RIR, reduced irrigation; N0, N1, N2, N3, and N4, N fertilizer addition were 0, 70, 140, 210, and 280 kg N/ha, respectively; *, ** The significant differences at *p* < .05 and *p* < .01, respectively; ns, no significant differences; The different letters indicate significant differences at the same irrigation level [*p* < .05])

**Figure 2:**
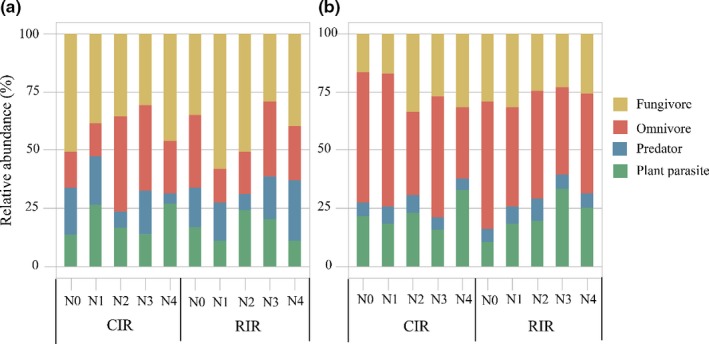
The relative abundance of the four soil mite trophic groups under reduced irrigation and nitrogen addition treatments in 2014 (a) and 2015 (b), respectively (CIR, conventional irrigation; RIR, reduced irrigation; N0, N1, N2, N3, and N4, N fertilizer addition were 0, 70, 140, 210, and 280 kg N/ha, respectively)

**Figure 3:**
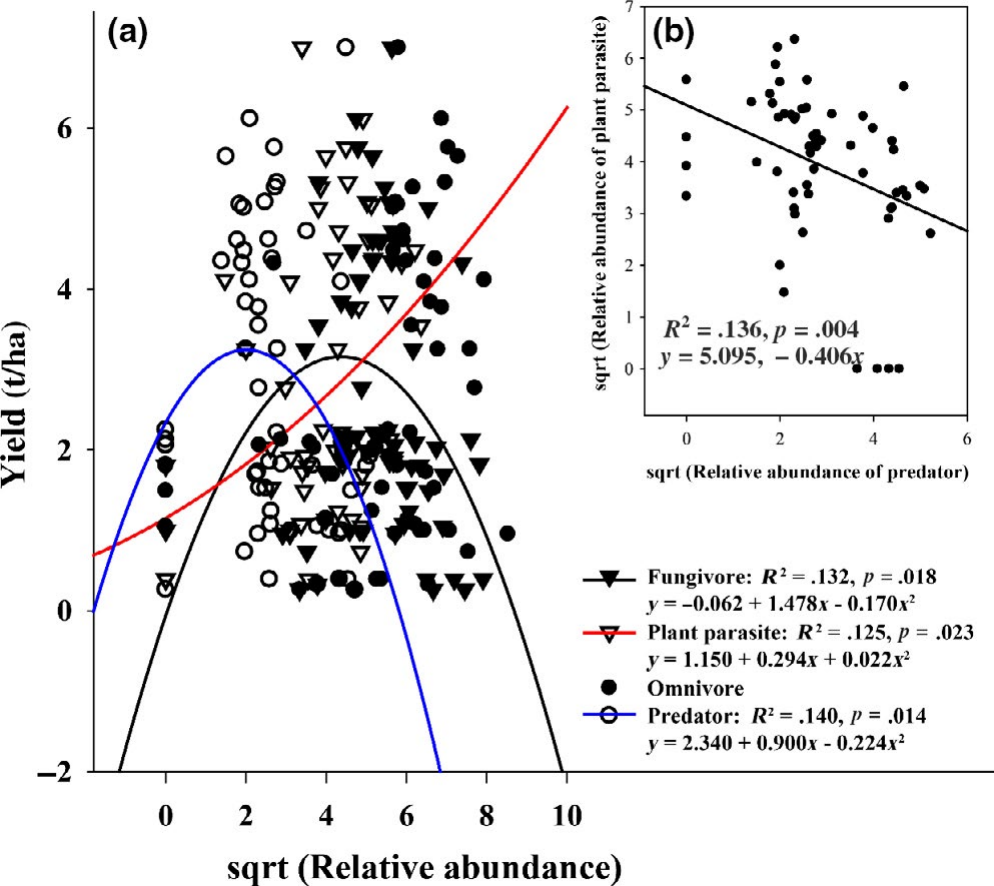
The correlations between the relative abundance of four soil mite trophic groups (fungivore, plant parasite, omnivore, and predator) and the wheat yields for reduced irrigation and nitrogen fertilizer addition treatments

Changes in nitrogen fertilizer addition significantly influenced wheat yields (Figure 1a,b; *p* < .05), whereas reduced irrigation addition and the interaction between reduced irrigation and nitrogen fertilizer addition showed no effects on wheat yield (Figure 1a,b; *p* > .05). Compared with N4, the reduced nitrogen fertilizer treatments (N3, N2, N1, and N0) changed wheat yield by −83.9% to 3.4% in 2014 and −80.4% to 16.6% in 2015. In addition, the wheat yields were similar in the N2, N3, and N4 application treatments, with the highest production in N3 treatments, while yield was significantly lower in the N1 and N0 application treatments than in the treatments with the other three levels of nitrogen fertilizer addition. Reduced irrigation and nitrogen fertilizer addition and their interactions did not alter the total abundances of soil mites (Figure 1c,d) or the relative abundances of mite trophic groups (Figure 2). Wheat yield exhibited quadratic relationships with the relative abundance of all mite trophic groups except omnivores (Figure 3). Specifically, an inverted bell‐shaped relationship was observed between the yield and the relative abundance of plant parasites (*y* = 0.022*x* ^2^ + 0.294*x* + 1.150; *R* ^2^ = .125, *p* = .023), a bell‐shaped relationship between yield and the fungivore relative abundance (*y* = −0.170*x* ^2^ + 1.478*x*−0.062; *R* ^2^ = .132, *p* = .018), and a bell‐shaped relationship between yield and predator relative abundance (*y* = −0.224*x* ^2^ + 0.900*x* + 2.340; *R* ^2^ = .140, *p* = .014), respectively (Figure 3a). We also found a significantly negative linear relationship between the relative abundances of plant parasites and predators (*y* = −0.406*x* + 5.095; *R* ^2^ = .136, *p* = .004; Figure 3b).

### 3.2 Community composition of soil mite under reduced irrigation and nitrogen fertilizer addition treatments

The reduced irrigation and nitrogen fertilizer addition treatments significantly altered the relative abundances of some mite taxa (Table 1). At the taxon level, a total of 18 families were identified in this study, which contained five fungivores, two plant parasites, five omnivores, and six predators. The fungivores and omnivores were the dominant groups in all samples and together accounted for 49.6%–73.0% and 64.1%–71.6% of all soil mites in 2014 and 2015, respectively. There were trade‐off correlations between the plant parasites with relative abundances among 7.9%–24.1% and predators with relative abundances of 3.9%–22.8%. The nymphs accounted for 1.2%–19.4% of all soil mite among the different treatments.

**Table 1:** Variations in significance in reduced irrigation (IR) and nitrogen fertilizer (N) addition, and their interaction (IR:N) for soil mite community composition

	2014						2015					
	IR		N		IR:N		IR		N		IR:N	
	*F*‐value	*p*‐value	*F*‐value	*p*‐value	*F*‐value	*p*‐value	*F*‐value	*p*‐value	*F*‐value	*p*‐value	*F*‐value	*p*‐value
Fungivore												
Scheloribatidae: *Scheloribates*	0.752	.397	0.452	.770	1.039	.414	0.000	.984	0.859	.507	0.421	.792
Ceratozetidae: *Ceratozetes*	1.384	.255	0.606	.663	0.276	.890	2.912	.105	1.849	.163	0.319	.861
Damaeidae: *Bella*	0.129	.724	1.249	.326	0.642	.640	0.017	.899	0.549	.702	**3.431**	**.030**
Oribatellidae: *Oribatella*	0.252	.622	2.332	.095	2.134	.118	0.025	.876	2.238	.105	0.504	.734
Microdispidae	1.179	.292	2.549	.075	**7.125**	**.001**	0.218	.646	1.919	.151	1.623	.212
Plant parasite												
Epilohmanniidae: *Epilohmannia*	0.008	.931	0.682	.614	0.483	.748	0.004	.953	0.523	.720	0.289	.881
Tetranychidae	0.338	.568	1.240	.329	0.143	.964	0.013	.912	2.673	.066	**3.435**	**.030**
Omnivore												
Oribatulidae: *Truncopes*	0.120	.733	0.946	.460	1.117	.379	0.491	.492	0.157	.957	0.898	.486
Zetorchestidae: *Zetorchestes*	0.015	.903	0.399	.807	1.801	.173	0.211	.651	2.383	.090	2.123	.120
Suctobelbidae: *Allosuctobelba*	2.069	.168	0.686	.611	**3.269**	**.035**	0.005	.946	0.832	.522	2.247	.104
Trhypochthoniidae: *Trhypochthonius*	0.091	.767	1.973	.142	0.900	.485	0.426	.522	0.984	.441	0.767	.560
Acaridae	**6.280**	**.022**	1.851	.163	0.203	.933	0.028	.870	0.546	.704	0.134	.968
Predator												
Scutacaridae	1.870	.188	2.352	.093	1.330	.297	3.425	.081	1.823	.168	1.234	.332
Stigmaeidae	0.216	.647	2.028	.133	1.025	.421	1.188	.290	1.204	.343	3.314	.034
Phytoseiidae	1.361	.259	0.926	.470	0.662	.627	0.244	.628	1.279	.315	0.017	.999
Laelapidae	**4.933**	**.039**	0.594	.672	1.213	.340	0.078	.784	0.504	.734	1.673	.200
Digamasellidae	3.150	.093	2.044	.131	1.865	.161	0.008	.932	2.214	.108	1.882	.157
Dermanyssidae	1.284	.272	0.462	.763	0.351	.840	0.278	.604	0.834	.521	0.893	.488
Total nymph	0.008	.929	3.500	.028	0.566	.690	0.017	.899	2.142	.117	1.300	.307

Note

Bold font indicates the significant effects of IR, N and IR:N.

**Figure 4:**
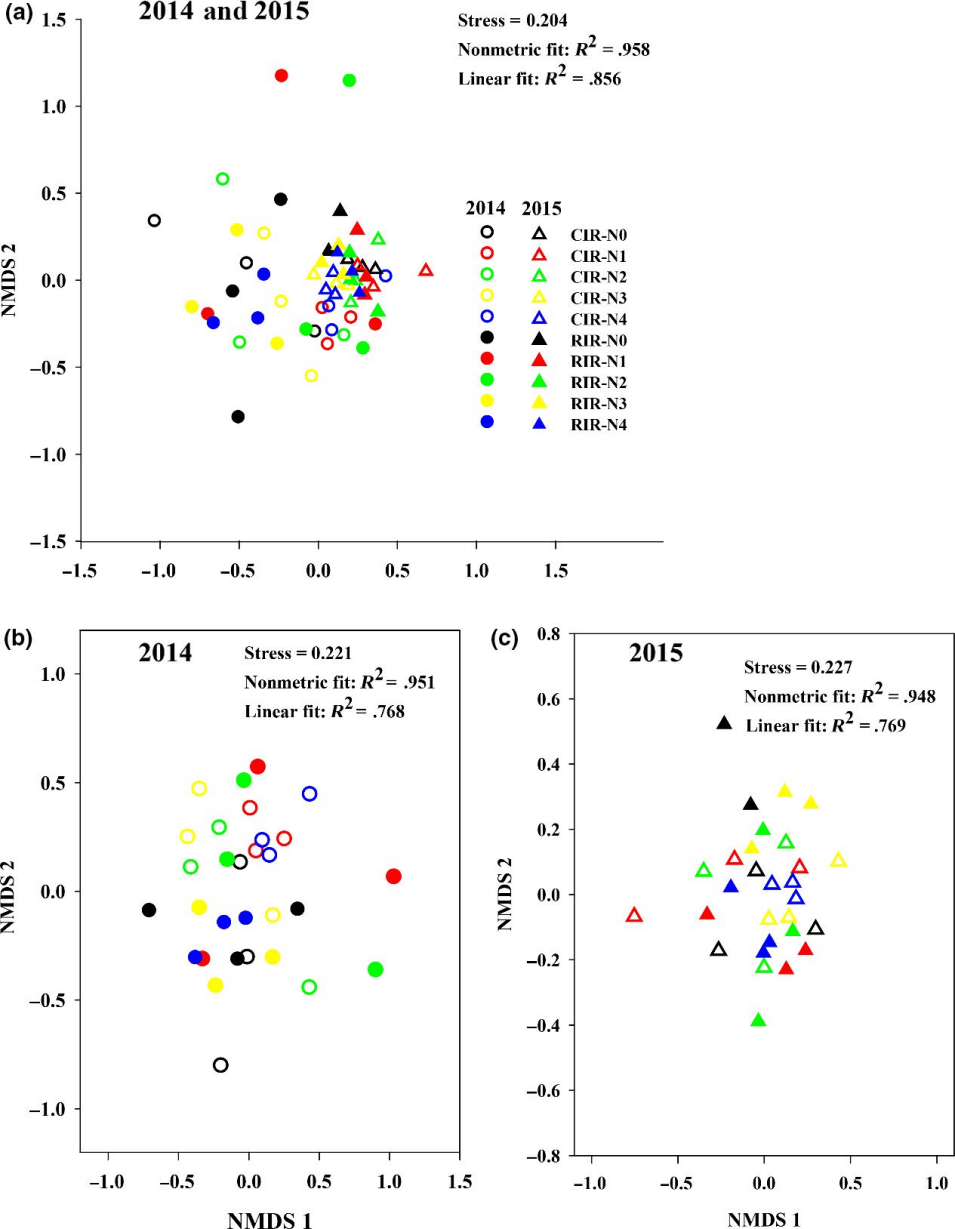
Nonmetric multidimensional scaling plot of the Bray–Curtis dissimilarity matrix of the soil mite community composition for the samples in combining 2014 and 2015 (a), 2014 (b), and 2015 (c), respectively (CIR, conventional irrigation; RIR, reduced irrigation; N0, N1, N2, N3, and N4, N fertilizer addition were 0, 70, 140, 210, and 280 kg N/ha, respectively)

**Figure 5:**
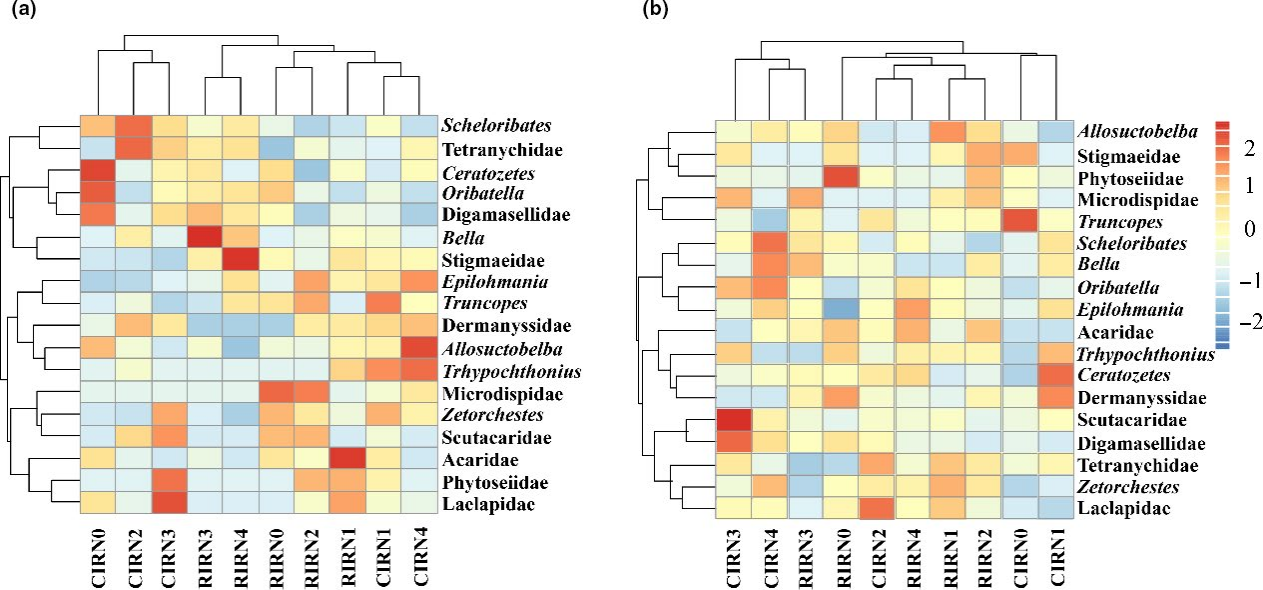
Distribution heatmaps of soil mite community composition based on the scaled relative abundance of each taxon under reduced irrigation and nitrogen fertilizer addition treatments in 2014 (a) and 2015 (b) (A higher abundance is shown in a darker color; treatments and mite taxa are ordered based on the hierarchical clustering of the abundance patterns [trees]; CIR, conventional irrigation; RIR, reduced irrigation; N0, N1, N2, N3, and N4, N fertilizer addition were 0, 70, 140, 210, and 280 kg N/ha, respectively)

The NMDS analysis, which was based on the relative abundances of mite taxa, showed that the soil mite community composition was first grouped by sampling year (Figure 4a, stress = 0.204) and was then divided by irrigation addition for a particular year (Figure 4b,c; stress = 0.221 and 0.227, respectively). The heatmaps showed the relative abundances of soil mite taxa under reduced irrigation and nitrogen fertilizer addition treatments in 2014 (Figure 5a) and 2015 (Figure 5b). The mite community hierarchical clustering showed that the relative abundance data were not well grouped by either irrigation addition or nitrogen fertilizer addition, except the samples from reduced irrigation treatment in 2014. Further analysis of the data using split‐plot ANOVA revealed that the reduced irrigation treatments decreased the relative abundance of Laelapidae but increased that of Acaridae in 2014 compared with conventional irrigation treatments; the combination of irrigation and nitrogen fertilizer addition had different effects on the relative abundances of *Allosuctobelba*, *Bella*, Tetranychidae, and Stigmaeidae (Table 1).

### 3.3 Relationship between soil environmental parameters and mite community composition

**Figure 6:**
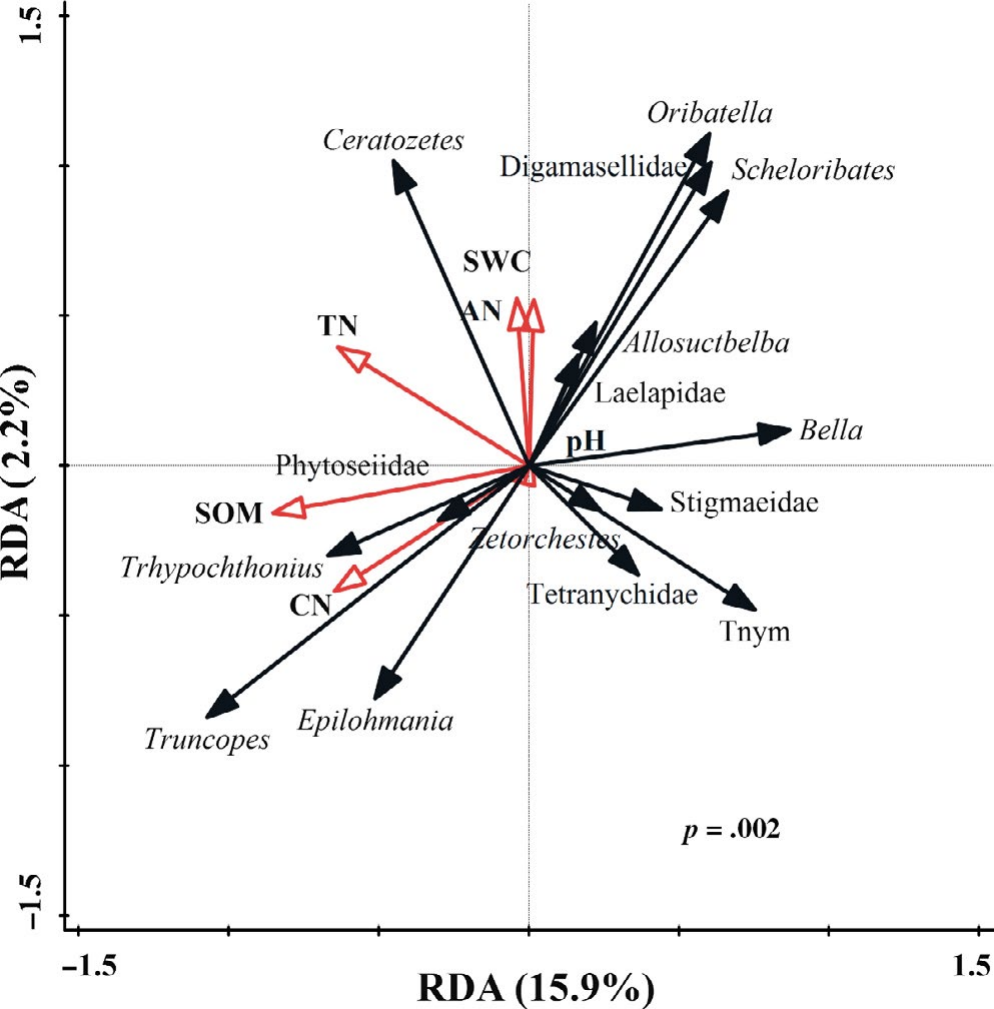
Redundancy analysis of soil properties and soil mite community composition (AN, available nitrogen; CN, carbon and nitrogen ratio; SOM, soil organic matter; SWC, soil water content; TN, total nitrogen)

According to the Mantel test and partial Mantel test, the soil mite community composition showed significant correlations with sampling year and soil environmental parameters but no correlations with reduced irrigation addition or reduced nitrogen fertilizer application (Table 2). Redundancy analysis (RDA) revealed that the variation in the soil mite community composition was significantly correlated with soil environmental parameters based on Monte Carlo test (*F* = 2.479, *p* = .002; Figure 6). The RDA ordination plot clearly demonstrated the relationships between soil environmental parameters and soil mite community composition, which had eigenvalues of 0.159 for the first axis and 0.022 for the second axis. The vectors showed that SOM, TN, and the CN ratio play a great role than SWC, pH, and AN in driving soil mite community composition. Stepwise regression further indicated that the soil mite community composition was mainly explained by SOM, TN, and the CN ratio (Table 3). In particular, the relative abundances of *Epilohmania* (Epilohmanniidae), Stigmaeidae, Laelapidae, and Digamasellidae were correlated with only the CN ratio, and the relative abundance of *Ceratozetes* (Ceratozetidae) was correlated with only TN. The relative abundances of *Truncopes* (Oribatulidae), *Trhypochthonius* (Trhypochthoniidae), and total nymphs could be explain by the SOM, TN, and the CN ratio, and the relative abundance of *Bella* was correlated with TN and the CN ratio. Furthermore, the relative abundance of Tetranychidae was correlated with SWC, pH, and the CN ratio, while that of Scutacaridae was correlated with both the SWC and the CN ratio.

**Table 2:** Mantel test and partial Mantel test of sampling year, reduced irrigation, and nitrogen fertilizer addition, and soil environmental parameters on soil mite community composition

	Mantel		Partial Mantel	
	Mantel score (*r*)	*p* value	Mantel score (*r*)	*p* value
Sampling year	**.393**	**.001**	**.392**	**.001**
Reduced irrigation	−.003	.513	.001	.400
Reduced nitrogen fertilizer	−.032	.757	−.030	.735
Soil properties	**.158**	**.035**	**.157**	**.034**

Note

Bold font indicates the significant mantel score (r).

**Table 3:** Variables responsible for the changes in soil mite community composition

	Result	*R* ^2^	*F*	*p*
Fungivore				
Ceratozetidae: *Ceratozetes*	*y* = −48.29 + 58.07 (TN)	.108	8.142	.006
Damaeidae: *Bella*	*y* = 73.24−3.04 (TN)−42.76 (CN)	.195	8.156	.001
Plant parasite				
Epilohmanniidae: *Epilohmania*	*y* = −34.02 + 6.53 (CN)	.126	9.536	.003
Tetranychidae	*y* = −101.52−0.30 (SWC) + 18.20 (pH)−31.40 (CN)	.214	6.343	.001
Omnivore				
Oribatulidae: *Truncopes*	*y* = −1,737.02−107.40 (SOM) + 1,486.73 (TN) + 220.33 (CN)	.282	8.739	<.001
Trhypochthoniidae: *Trhypochthonius*	*y* = −177.83−11.27 (SOM) + 151.05 (TN) + 23.18 (CN)	.186	5.482	.002
Predator				
Scutacaridae	*y* = 85.62−9.73 (SWC)−1.05 (CN)	.075	3.408	.040
Stigmaeidae	*y* = 15.10−1.80 (CN)	.057	4.556	.037
Laelapidae	*y* = −8.75 + 0.22 (CN)	.079	6.088	.017
Digamasellidae	*y* = 26.08−3.16 (CN)	.120	9.040	.004
Total nymph	*y* = 886.55 + 53.96 (SOM)−755.63 (TN)−108.51 (CN)	.222	6.606	.001

Abbreviations: CN, carbon and nitrogen ratio; SOM, soil organic matter; SWC, soil water content; TN, total nitrogen.

## 4 DISCUSSION

Our results showed that wheat yields were not significantly decreased by moderately reduced nitrogen fertilizer addition (280–140 kg N/ha at increments of 70 kg N/ha), and maximum yields were obtained with 210 kg N/ha except under conventional irrigation treatments in 2014. The effect of reduced nitrogen fertilizer addition on wheat yields was similar to that in a previous data analysis in which the maximum yield was achieved at approximately 210 kg N/ha in NCP (Chen et al., 2014; Liu et al., 2016). This result suggests that an appropriate decline in nitrogen fertilizer addition will not reduce the wheat grain yield but will increase nitrogen partial factor productivity and reduce environmental hazards (Chen et al., 2014; Ju et al., 2009). The reduced irrigation treatments (20% lower than conventional irrigation addition) did not have a more significant negative effect on wheat grain production than the conventional irrigation treatments. The wheat yields in the reduced irrigation treatments were the same (2014) or higher (2015) than those in the conventional irrigation treatments. Similar results were obtained in previous studies in which reduced irrigation addition saved water use and advanced the wheat flowering date, which was helpful to prevent agricultural disaster (e.g., dry hot wind) and increasing yield (Meng et al., 2012; Wang et al., 2016, 2018).

According to the abovementioned positive effects of reduced irrigation and nitrogen fertilizer addition on grain yield, our study demonstrated the response of soil mite community composition to reduced irrigation and nitrogen fertilizer addition treatments in two continuous sampling years in a long‐term field trial. The reduced irrigation treatments increased the relative abundances of Acaridae and Laelapidae, suggesting that soil water content was important in affecting soil mite abundance (Olear & Blair, 1999). This finding was consistent with that a previous study demonstrated that mites were less abundant in irrigated plots (Olear & Blair, 1999). In contrast, the results of a short‐term experiment showed that irrigation had no effects on mite abundance (Liu, Li, Liu, & Yang, 2017; Pressler, Foster, Moore, & Cotrufo, 2017), whereas in other treatments with increased water addition in grassland ecosystems, there were significant positive effects on mite communities (Chikoski, Ferguson, & Meyer, 2006; Wu et al., 2014). The inconsistent impacts of irrigation addition on soil mites suggested that the responses of mite to changes in irrigation addition are independent of taxa and the duration and vegetation type in the experiment (Blankinship, Niklaus, & Hungate, 2011). Our results also revealed that reduced irrigation and nitrogen fertilizer addition had interactive effects on the relative abundances of Microdispidae, *Allosuctobelba* (Suctobelbidae), *Bella* (Damaeidae), and Tetranychidae. A previous study found that low‐dose nitrogen fertilizer in combination with irrigation had slightly positive effects on mites (Lindberg & Persson, 2004). Few studies have investigated the response of soil mite community to combined irrigation and nitrogen fertilizer addition. Therefore, more relevant studies are needed to evaluate the specific response of soil mites to reduced irrigation and nitrogen fertilizer addition in various ecosystems.

In the present study, soil mite community structure was grouped separately by sampling year, and the mite abundance was lower in 2014 than in 2015. We suspected that the decline was correlated with the lack of resources available in soil (e.g., debris and exudates from wheat) to mites in wheat field. The growth of wheat is influenced by multiple factors, such as precipitation, soil fertility, and climate disasters (Telfer et al., 2018; Zhang, Wang, & Niu, 2019). The effective precipitation was 14.3% lower in 2014 than in 2013, and hot dry wind occurs in early May of 2014 (local meteorological data), which had adverse effects on wheat growth. Root exudates are affected by crop growth and soil water availability (Maurer, Kiese, Kreuzwieser, & Rennenberg, 2018), which subsequently influenced the soil mite community (Lemanski & Scheu, 2014; Strickland, Wickings, & Bradford, 2012).

The impacts of reduced irrigation and nitrogen fertilizer addition on soil mite taxa distribution were presented by heatmaps in this study. The distribution of soil mites was inconsistent between 2014 and 2015, which suggested that the soil mite community may not be directly influenced by the irrigation and nitrogen fertilizer addition. Multiple studies have shown that soil properties shape the soil mite community structure (Cao et al., 2011; Nielsen, Osler, Campbell, Burslem, & Van Der Wal, 2010; Wang et al., 2015). Our results revealed that soil environmental parameters (e.g., SOM and TN) significantly influenced soil mite community composition. RDA analysis and stepwise regression analysis were used to explore the influences of soil properties and to reveal the factors that affected the soil mite taxa. We found that SOM, TN, and the CN ratio strongly influenced the soil mite community, an effect that was previously explained by supplying of food resources (Taylor & Wolters, 2005; Wickings & Grandy, 2011). This finding is consistent with previous studies in which SOM explained a significant part of the variation in soil mite community distribution (Gao, He, Zhang, Liu, & Wu, 2014). Reduced irrigation and nitrogen fertilizer addition had no effects on the soil properties and soil mite community in this study, whereas the SOM, TN, and the CN ratio had significant effects on the relative abundance of soil mite taxa, both individually and in combination. Based on previous studies (Wang et al., 2015; Wu et al., 2014), we suspect that reduced irrigation and nitrogen fertilizer addition primarily changed soil mite community by affecting SOM, TN, and the CN ratio. To clarify the influence of reduced irrigation and nitrogen fertilizer addition on soil mite community composition, more relevant studies should be conducted in the future.

We explored the potential correlations between soil mite trophic groups and wheat yields, which revealed the interactive relationships between the mite community composition and wheat yields. Soil mite community could be influenced by plant growth, as plants provide habitats and/or resources for soil organism through inputs of chemical compounds and organic matters (Bardgett & Wardle, 2010). In this study, the mite trophic groups had significant nonlinear relationships with wheat yields, suggesting that the effects of soil mites on crops may through indirect pathways (Dirilgen et al., 2016; Ito, 1971; Maraun et al., 2014). Previous studies showed that fungivorous mites and parasitic mites contributed to the decomposition of organic matter in the soil from roots and other organic sources for soil nutrients releasing and activation (Siepel & Maaskamp, 1994), and the feeding activity of fungivores plays a key role in increasing soil aggregate stability and soil formation (Maaß et al., 2015). The effects of soil mite trophic groups on wheat yields maybe mainly indirectly change soil nutrients or regulate other soil organism community (Koehler, 1999; Maraun et al., 2014). Additional, plant growth was influenced by management practices, biotic and abiotic factors, the effects of soil mite on wheat yields are also lack of relevant researches. Therefore, it is necessary to conduct more studies to evaluate the effects of soil mite and combined other soil organism on wheat yields.

## 5 CONCLUSIONS

This study explored the effects of the combined reduction in irrigation and nitrogen fertilizer addition on wheat grain yield and the soil mite community composition in a long‐term field trial. The results indicated that reasonable irrigation and nitrogen fertilizer reduction will not decrease the wheat yields or impact the soil mite community composition, which were 20% and 25% lower, respectively, than those under conventional irrigation and nitrogen fertilizer addition. Wheat yields were affected by changes in the relative abundances of fungivores, plant parasites, and predators. Soil mite community taxa were more directly responsive to changes in soil environmental parameters than to irrigation and nitrogen fertilizer addition. Our results suggest that the impacts of reduced irrigation and nitrogen addition on soil mite taxa may occur indirectly through changes in soil environmental parameters.

## CONFLICT OF INTEREST

None declared.

## AUTHOR CONTRIBUTIONS

CYZ identified soil mite, analyzed the data, and wrote the paper. FG and FOY designed and oversaw the study. FOY, CYZ, and XHL did field sampling. ZOY, JHM, and FHZ assisted with the field work.

## Data Availability

We agree to make our data publicly available in a relevant repository. Experimental data (soil mite morphological data): https://doi.org/10.5061/dryad.50dh670
